# Targeted Single-Walled Carbon Nanotubes for Photothermal Therapy Combined with Immune Checkpoint Inhibition for the Treatment of Metastatic Breast Cancer

**DOI:** 10.1186/s11671-020-03459-x

**Published:** 2021-01-07

**Authors:** Patrick McKernan, Needa A. Virani, Gabriela N. F. Faria, Clément G. Karch, Ricardo Prada Silvy, Daniel E. Resasco, Linda F. Thompson, Roger G. Harrison

**Affiliations:** 1 Department of Neurology, Neurosurgery, and Radiation Oncology, University of Oklahoma Health Sciences Center, Oklahoma City, OK USA; 2 Department of Radiation Oncology, Brigham and Women’s Hospital, Harvard Medical School, and Dana-Farber Cancer Institute, Boston, MA USA; 3 School of Chemical, Biological and Materials Engineering, University of Oklahoma, Norman, OK USA; 4 School of Biomedical Engineering, University of Oklahoma, Norman, OK USA; 5 Arthritis and Clinical Immunology Program, Oklahoma Medical Research Foundation, Oklahoma City, OK USA; 6 Stephenson Cancer Center, Oklahoma City, OK USA

**Keywords:** Single-walled carbon nanotubes, Photothermal therapy, Annexin A5, Breast cancer, Immune checkpoint inhibitor

## Abstract

The greatest contributors to cancer mortality are metastasis and the consequences of its treatment. Here, we present a novel treatment of metastatic breast cancer that combines photothermal therapy with targeted single-walled carbon nanotubes (SWCNTs) and immunostimulation with a checkpoint inhibitor. We find that the selective near-infrared photothermal ablation of primary orthotopic EMT6 breast tumors in syngeneic BALB/cJ mice using an annexin A5 (ANXA5) functionalized SWCNT bioconjugate synergistically enhances an anti-cytotoxic T-lymphocyte-associated protein 4 (anti-CTLA-4)-dependent abscopal response, resulting in an increased survival (55%) at 100 days after tumor inoculation. In comparison, there was no survival at 100 days for either photothermal therapy by itself or immunostimulation by itself. Prior to photothermal therapy, the SWCNT-ANXA5 bioconjugate was administered systemically at a relatively low dose of 1.2 mg/kg, where it then accumulated in tumor vasculature via ANXA5-dependent binding. During photothermal therapy, the average maximum temperature in the tumor reached 54 °C (duration 175 s). The mechanism of prolonged survival resulting from combinatorial photothermal ablation and immune stimulation was evaluated by flow cytometric quantification of splenic antitumoral immune effector cells and serum cytokine quantification.

## Introduction

Metastasis and the consequences of its treatment are the single greatest cause of death in cancer [1]. For example, when breast cancer metastasizes, patient 5-year survival rates fall below 25%. Even though in the last 60 years over 200 new antineoplastic drugs have improved patient outcomes, overall survival remains poor in metastatic disease [2]. Here, a novel combination of photothermal therapy facilitated by a tumor targeted singe-walled carbon nanotube (SWCNT) bioconjugate and anti-cytotoxic T-lymphocyte-associated protein 4 (anti-CTLA-4) checkpoint inhibition is studied for treating metastatic breast cancer in an orthotopic murine model.

The unique properties of SWCNTs as a nanomaterial have generated significant interest in their use as a potential tool in the fight against cancer. While SWCNTs exert a variety of biological effects, the use of SWCNTs in the treatment of cancer has focused primarily on their interaction with near-infrared (NIR) light and the resulting photothermal effect, where SWCNTs rapidly heat the tumor in the process called photothermal therapy (PTT). Numerous groups have investigated the potential for SWCNTs to be used in PTT-based treatment strategies in several models of breast cancer [3–10]. These works have focused largely on the ability of PTT to treat primary tumors at depths no greater than several mm, where the attenuation of NIR light is almost complete.

Earlier, we showed that primary orthotopic breast tumors in syngeneic mice could be nearly completely eliminated using mild NIR laser light in conjunction with a photothermal enhancing SWCNT bioconjugate [11]. In this bioconjugate, the SWCNTs were functionalized with the protein annexin A5 (ANXA5), which binds with high affinity to the anionic phospholipid phosphatidylserine expressed externally on tumor cells and on endothelial cells of the tumor vasculature, but not on normal cells in the vasculature [12–14]. This conjugate was visualized using atomic force microscopy (AFM) to show that the height of the conjugate was between 2.5 and 5.0 nm, which is similar to that for other SWCNT-protein conjugates [11]. While capable of eradicating primary tumors in this previous metastatic model, photothermal therapy alone only modestly extended survival. However, we found preliminary evidence suggesting that concomitant treatment with immunomodulators such as cyclophosphamide was capable of increasing survival.

Recently, the potential for immunomodulators such as cyclophosphamide to synergistically enhance SWCNT directed PTT has been the subject of much interest. One category of promising immunomodulating agents are checkpoint inhibitors. Checkpoint inhibitors are antibodies such as anti-CTLA-4, anti-PD-1, and anti-PDL-1 which bind critical cell proteins responsible for modulating the body’s response to cancer. These antibodies block key biological “checkpoints” where the body can down-regulate the immune system’s response to cancer. These proteins normally serve an important role in controlling the body’s immunological response. By blocking the action of these proteins, checkpoint inhibitors remove a mechanism by which the immune system normally suppresses its natural antitumoral response. Recently, several groups have observed that the combination of anti-CTLA-4 check point inhibition with SWCNT enhanced PTT has the potential to promote a robust immune response in breast cancer [15, 16].

In the current study, we evaluate the combination of our novel PTT modality in conjunction with the immunostimulatory agent anti-cytotoxic T-lymphocyte-associated protein 4 (anti-CTLA-4). Originally approved for the treatment of metastatic melanoma [17], anti-CTLA-4 is now being tested for treating breast cancer in clinical trials combined with other immunostimulatory agents [18]. We evaluate the mechanism of enhanced antineoplastic immunity in PTT in combination with anti-CTLA-4 checkpoint inhibition, as well as the longer term fate of SWCNTs in target organs.

## Materials and Methods

### Materials

The plasmid encoding ANXA5, pET-30 Ek/LIC/ANX, was previously constructed in this laboratory [11]. Bovine serum albumin (BSA), Triton X-100, EDTA, β-mercaptoethanol, phenylmethylsulfonyl fluoride (PMSF), and Tris–acetate-EDTA buffer were from Sigma-Aldrich (St Louis, MO). Sodium phosphate and sodium dodecyl sulfate (SDS) were from Mallinckrodt Chemicals (Phillipsburg, NJ). HPLC grade ethanol was from Acros Organics (Waltham, MA). His-trap columns were from GE Healthcare (Chicago, IL). Flow cytometry staining buffer, fixation/permeabilization buffer, permeabilization buffer, chromogenic endotoxin quantification kit, and Slide-A-Lyzer dialysis cassettes (3.5 kDa) were from Thermo Fisher Scientific (Waltham, MA). The 2 and 100 kDa dialysis membranes were from Spectrum Laboratories (Rancho Dominguez, CA). Roswell Park Memorial Institute cell medium (RPMI-1640) and Hank’s balanced salt solution were from ATCC (Manassas, VA). Fetal bovine serum (FBS) was from Atlanta Biologicals (Lawrenceville, GA). Tryptone, yeast extract, and kanamycin monosulfate were obtained from Alfa Aesar (Ward Hill, MA). Sodium hydroxide, potassium chloride, and sodium chloride were from VWR Inc (Radnor, PA). HRV-C3 protease was from Sino Biologics (Portland, OR). Anti-CTLA-4 mouse monoclonal antibody (clone: 9H10) and mouse cytokine ELISA kits (TNF-α, IFN-γ, IL-6) were obtained from BioLegend (San Diego, CA). CoMoCAT SWCNTs (average diameter 0.8 ± nm, average length 1.5 ± 0.5 µm) were obtained from CHASM (Boston, MA). The CoMoCAT method is known to yield SWCNT of a small number of (*n*,*m*) chiralities with high selectivity [19]. The sample used in this study is highly enriched in [5, 6] SWCNTs, which exhibits a strong NIR light absorption at a wavelength of 980 nm. This absorption corresponds to the S_11_ transition for this type of nanotube between an occupied van Hove singularity to the corresponding unoccupied one. Therefore, to maximize the radiation absorption by the SWCNT deposited on the tumors, the wavelength of the laser used in this study was 980 nm to exactly match that of the S_11_ optical transition [20]. Figure S1 in the Additional file 1 shows the fluorescence spectra, clearly showing the S_11_ NIR emission after exciting the S22 transition with visible light.

### Cell Culturing

EMT6 breast cancer cells from ATCC (Manassas, VA) were cultured in Waymouth’s MB 752/1 medium with 2 mM L-glutamine supplemented with 15% FBS. All cells were grown at 37 °C and 100% humidity under 5% CO_2_. All cells were passaged using 0.25% (w/v) trypsin in 0.53 mM EDTA. Cell lineage and mycoplasma free status were confirmed via STR testing (Charles River) and assayed to be endotoxin free by limulus assay.

### ANXA5 and SWCNT-ANXA5 Production

The SWCNT-ANXA5 conjugate was prepared using a procedure we previously developed that gives 2.5 mg ANXA5/mg SWCNT [11]. Briefly, *E. coli* transfected with a plasmid encoding ANXA5, pET-30 Ek/LIC/ANX, were grown and purified using immobilized metal affinity chromatography with immobilized Ni^2+^ to isolate the ANXA5, which involved enzymatic cleavage to remove a (His)_6_ tag. Freeze-dried CoMoCAT SWCNTs were dispersed in 1% sodium dodecyl sulfate (SDS) using two cycles of probe sonication at 20 W and centrifugation at 29,600 g for 30 min each. The suspended SWCNTs were characterized by NIR fluorescence (Additional file 1: Figure S1) and then conjugated to a 1,2-distearoyl-sn-glycero-3-phosphoethanolamine-polyethylene glycol-maleimide (DSPE-PEG-maleimide) linker for 30 min at room temperature to allow hydrophobic interaction between SWCNTs and the DSPE functional group. This was then followed by an 8 h dialysis in distilled water to remove excess linker and SDS. The dialyzed conjugate was then reacted with ANXA5, which contains one cysteine group, for 2 h and blocked with 1.5 mg ml^−1^ L-cysteine. The final product, SWCNT-ANXA5, was dialyzed against 20 mM sodium phosphate buffer for 8 h to remove excess ANXA5 and L-cysteine. Protein weight and purity were characterized via SDS-PAGE. The SWCNT and ANXA5 content of the bioconjugate was characterized by UV–Vis–NIR fluorescence spectroscopy, Fourier-transform infrared spectroscopy (FT-IR) Raman analysis, and Bradford assay (Additional file 1: Figure S2).

### In Vivo Studies

All procedures complied with a protocol approved by Institutional Animal Care and Use Committee (IACUC) of the University of Oklahoma. Female BALB/cJ female mice 6 weeks of age were used (Jackson Laboratory, Bar Harbor, ME). Mice were fed a standard chow diet. During photothermal irradiation of tumors with a NIR laser light, mice were anesthetized with 2% isoflurane and 98% oxygen using a nose cone.

Tumors were induced by orthotopic injection of 10^6^ EMT6 mouse breast cancer cells in 100 µL of PBS into the IV mammary fat pad. Tumors were allowed to grow for 12 days, and when they reached a volume of 60 mm^3^ (~ 5 mm in diameter), mice received a systemic i.v. injection of 1.2 mg/kg of SWCNT-ANXA5 (mg SWCNT per kg body weight) bioconjugate via the lateral tail vein. After 3 h, a region 5 mm in excess of the tumor boundary was irradiated with NIR light (980 nm) at an energy and power level of 175 J/cm^2^ and 1 W/cm^2^, respectively (time of 175 s; Diodevet-50 NIR laser, B&W Tek Inc., Newark, DE). Checkpoint inhibition was accomplished by serial i.p. administration of 200 µg anti-CTLA-4 antibody in 100 µl of PBS on days 8, 11 and 16 following tumor inoculation. Tumor volume was calculated with the modified ellipsoid formula $V = \frac{1}{2} \times {\text{length}} \times {\text{width}}^{2}$ using caliper measurements of the longest dimension and perpendicular width. Tumor temperature was monitored by a handheld FLIR TG165 Spot thermal camera (Raymarine ITC, Fareham, UK) set to autoscan for maximum detected temperature. Target organs were harvested for toxicity evaluation and fixed in 10% neutral buffered formalin, and formalin-fixed-paraffin-embedded (FFPE) slides stained were prepared and stained with hematoxylin and eosin for toxicity analysis.

### Ex Vivo SWCNT Detection

Mice received a systemic injection of 1.2 mg/kg of SWCNT-ANXA5 (mg SWCNT/kg body weight) via the lateral tail vein. At select time points, mice (*n* = 3) were euthanized, necropsied, and target organs removed for analysis. Tissue samples were prepared as described previously [21]. The presence of SWCNTs in ex vivo tissue samples was then determined by relative NIR fluorescence spectroscopy using a NS3 NanoSpectralyzer (Applied NanoFluorescence, Houston, TX).

### Flow Cytometry

Mice were euthanized 14-day post-treatment, and splenic antitumoral immune effector cells were quantified as previously described [21].

### Cytokine Detection

Following treatment as described above, mice (*n* = 4–5) were euthanized 7 days after photothermal therapy for blood collection. The concentration of the tumor necrosis factor alpha (TNF-α), interferon gamma (IFN-γ), and interleukin 6 (IL-6) in diluted serum samples was quantified by ELISA according to the supplier’s protocol.

### Statistical Analysis

Data were analyzed with Graphpad Prism software. Statistical significance was assessed using a one-way ANOVA and Tukey–Kramer multiple comparisons test. Statistical significance of survival curves was determined by the Mantel–Haenszel log-rank test. Statistical significance of cytokine serum concentration was analyzed by one-way ANOVA and Dunnett's multiple comparisons test. Multiple comparisons were corrected by the Bonferroni threshold. Error is represented graphically as standard error of the mean unless error did not exceed the size of the plotted mean point symbol, in which case the bars were excluded for clarity.

## Results

### Thermal Kinetics

**Fig. 1:**
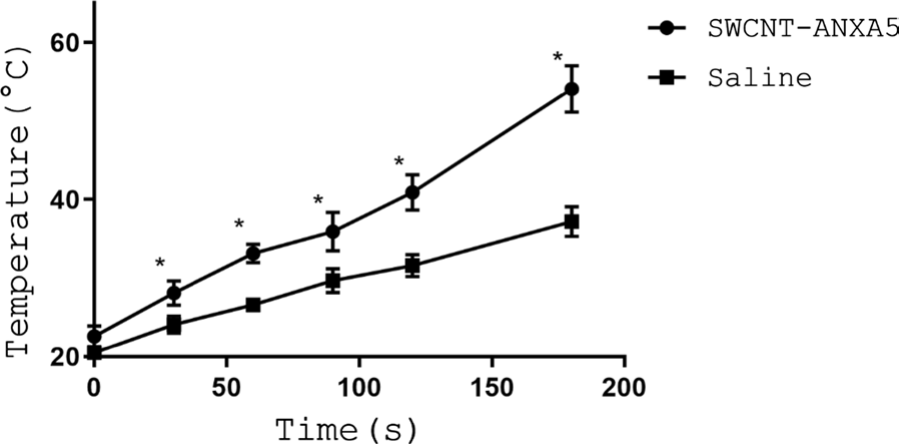
Thermal kinetics resulting from radiation of tumors with NIR light at 980 nm. BALB/cJ mice bearing EMT6 tumors were injected by i.v. in the tail vein with 1.2 mg/kg SWCNT-ANXA5. After waiting 3 h to allow clearance of SWCNTs from circulation, tumors were irradiated with a NIR laser at an energy and power density of 175 J/cm^2^ and 1 W/cm^2^, respectively (*t* = 175 s). The maximum temperature of the tumor was recorded as a function of time. The temperature of tumors from mice receiving SWCNT was significantly higher than for mice receiving physiological saline at *t* = 175 s (*p* < 0.05). Data are presented as mean ± SE (*n* = 3)

Using a dose of 1.2 mg/kg SWCNT-ANXA5, the maximum temperature of tumors was recorded over the course of treatment with NIR light, as indicated in Fig. 1. Mice that received SWCNT-ANXA5 prior to irradiation had higher tumor temperatures for the entire NIR light treatment compared to mice that received physiological saline. The average temperature of the tumor for mice receiving SWCNT-ANXA5 was significantly different than it was for mice receiving physiological saline (54 °C vs. 37 °C, *p* < 0.05). As a result of this enhanced photothermal therapy, only in the SWCNT-ANXA5 group did visible tumor ablation occur. Tumor ablation was characterized by rapid discoloration, followed by skin contracture and the appearance of wrinkles. Within 48 h, a significant eschar formed at the site of photothermal ablation. Complete skin regrowth took several weeks.

### Photothermal Therapy and Checkpoint Inhibition

**Fig. 2:**
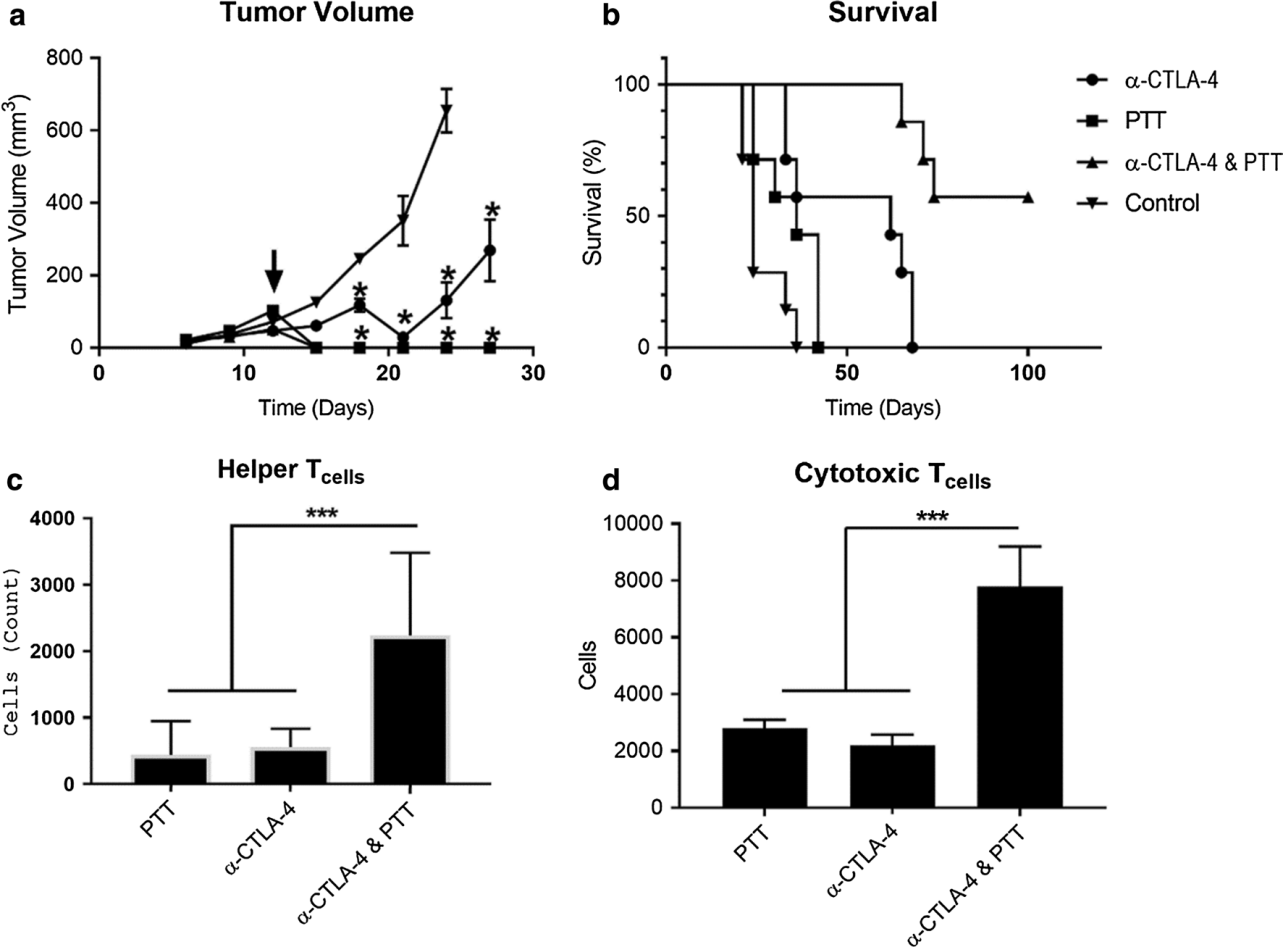
Results of combinatorial photothermal therapy (PTT) and checkpoint inhibition (anti-CTLA-4) in EMT6 tumors. Mice with well-developed orthotopic syngeneic tumors (*d* ≥ 5 mm) were administered an i.v. systemic dose of 1.2 mg/kg SWCNT-ANXA5. **a** Tumor volumes were then monitored following irradiation (arrow) with NIR laser light for 175 s at a power density of 1 W/cm^2^ on day 12 following inoculation. In addition to PTT, select groups received anti-CTLA-4 (100 µg) on days 8, 11, and 16. Mice in the control group were injected i.v. with physiological saline. Tumor volume shown as mean ± SE (*n* = 7). Significance compared to the control is indicated by * (*p* < 0.05). **b** The combination of photothermal therapy and immune checkpoint inhibition significantly increased survival compared to controls (*p* < 0.05, *n* = 7). **c**, **d** Only when mice received photothermal therapy in conjunction with anti-CTLA-4 checkpoint inhibition were significantly increased in the relative numbers of CD4^+^ and CD8^+^ splenocytes observed 2 weeks after PTT. Splenocytes shown as mean ± SE (*n* = 3). Significance is indicated by *** (*p* < 0.005)

The results of combining photothermal therapy with checkpoint inhibition using anti-CTLA-4 monoclonal antibody are shown in Fig. 2. While photothermal therapy alone readily eradicated primary breast cancer neoplasia, the inability of the NIR laser light to penetrate beyond a few mm limited the treatment of breast cancer metastasis. In order to overcome the shortfalls of localized NIR photothermal antineoplastic treatment, we explored the combination of this unique therapeutic treatment modality with systemic checkpoint inhibition (Fig. 2). While photothermal therapy exceled at destroying primary EMT6 tumors (Fig. 2a), this treatment failed to eradicate metastasis and only modestly increased the survival of mice bearing EMT6 orthotopic tumors (Fig. 2b). In contrast, checkpoint inhibition with anti-CTLA-4 enhanced overall survival but only temporarily delayed primary tumor growth. While neither therapy alone yielded any increases in overall survival, the combination of both SWCNT-ANXA5 enhanced photothermal therapy and anti-CTLA-4 checkpoint inhibition improved overall survival, leading to 55% survival at 100 days post-tumor inoculation.

Cytometric analysis of splenic effector cells following treatment revealed a putative mechanism of improved survival in mice receiving combinatorial photothermal therapy and checkpoint inhibition. Mice were inoculated with EMT6 and treated as described earlier. Two weeks after treatment, mice were sacrificed and both the percentages and relative number of multiple immune effector types were quantified. Populations of helper T-cells, cytotoxic T-cells, neutrophils, myeloid-derived suppressor cell (MDSC) monocytes, FoxP3 regulatory T-cells, and macrophages were evaluated (Additional file 1: Figure S3). Analysis of these populations revealed significant differences between treatment populations and controls only in mice which received the combination of both photothermal therapy and anti-CTLA-4 checkpoint inhibition together. In those animals which received this combination, we observed an increase in the relative numbers of helper T cells (CD4^+^) and cytotoxic T cells (CD8^+^), (Fig. 2c, d). Increases in CD4^+^ and CD8^+^ cell counts correlated with gross increases in spleen sizes observed following necropsy.

**Fig. 3:**
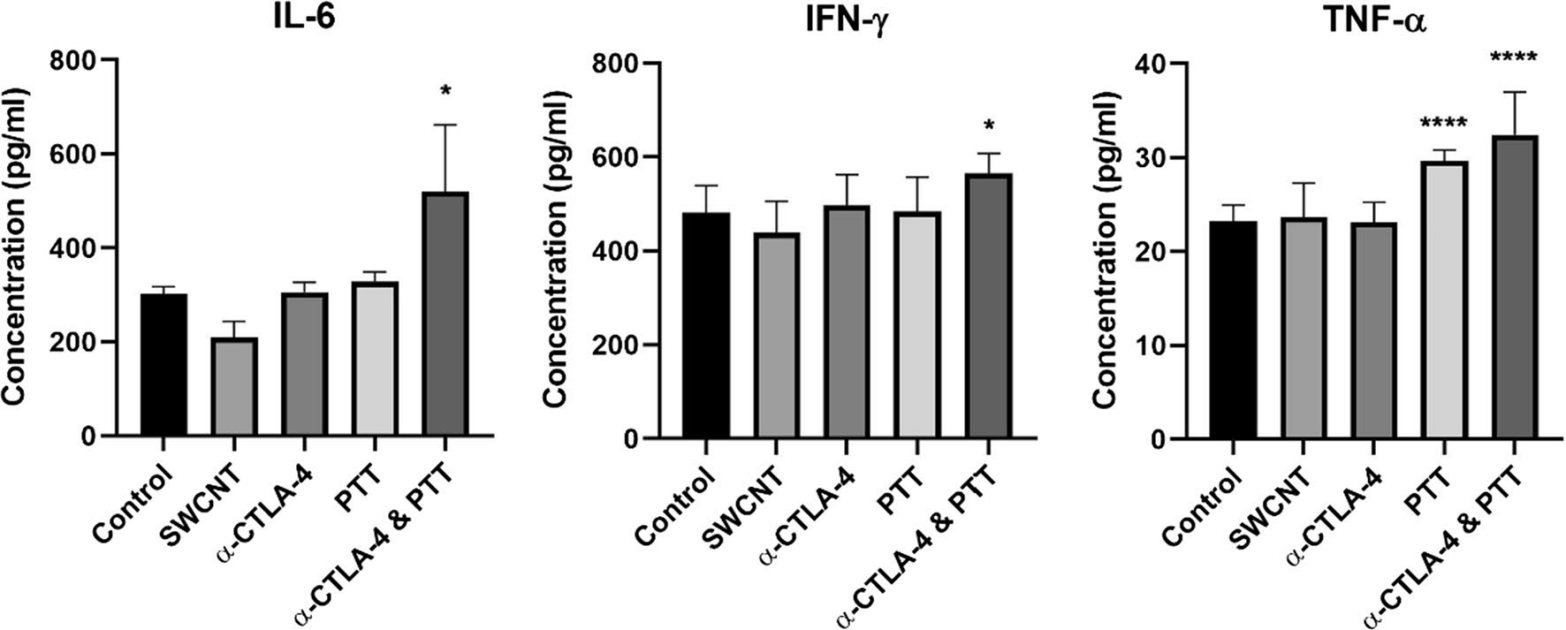
Serum cytokine concentration. Quantification of serum cytokine levels in mouse serum 7 days after PTT showed a significant increase of IL-6, IFN-γ, and TNF-α levels in mice following PTT in conjunction with check-point inhibition (anti-CTLA-4). The results are shown for the untreated control, SWCNT treatment only, anti-CTLA-4 treatment only, PTT treatment, and PTT + anti-CTLA-4 treatment. Data are presented as mean ± SE (*n* = 4–5). Statistical significance was analyzed for the treated groups compared to the untreated control group by one-way ANOVA with Dunnett's multiple comparisons test. Statistical significance is indicated by * (*p* < 0.05) and **** (*p* < 0.0001)

In addition to cytometric analysis of splenic effector cells, serum cytokine concentration was determined 7 days following PTT to help elucidate the mechanisms of antitumoral immunity. The levels of the pro-inflammatory cytokines IL-6, IFN-γ, and TNF-α are presented in Fig. 3. Neither SWCNT nor anti-CTLA-4 treatment alone significantly increased the levels of cytokines compared to the untreated control group. However, PTT treatment alone was able to increase TNF-α significantly. The further addition of anti-CTLA-4 to PTT treatment significantly increased the levels TNF- α, IL-6, and IFN-γ.

### Biodistribution and Toxicity of SWCNT-ANXA5

**Fig. 4:**
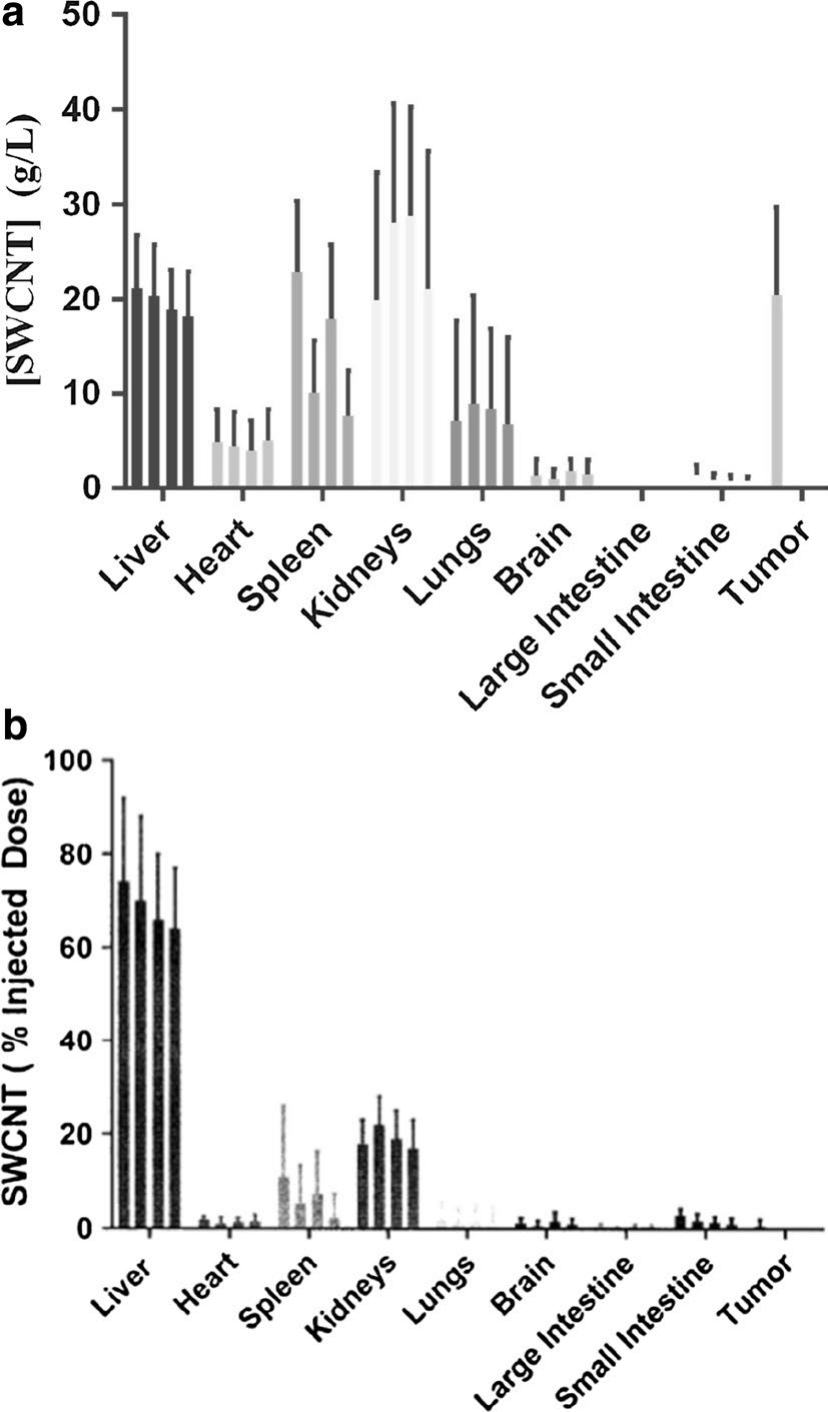
Biodistribution of SWCNT-ANXA5 measured as % of injected dose (**a**) and tissue concentration in g/L (**b**). The SWCNT-ANXA5 conjugate was i.v. injected into Balb/cJ mice at a dose of 1.2 mg/kg. The SWCNT concentration was measured in various organs of mice without tumors after 1, 2, 3, and 4 months (left to right). For mice with tumors, the concentration of SWCNTs was measured at the time as it would be immediately prior to photothermal therapy treatment. Data are shown as mean ± SE (*n* = 3)

The biodistribution of SWCNT-ANXA5 following administration for photothermal therapy enhancement in various organs was monitored with fluorescent quantification of SWCNT in ex situ tissue lysates compared to standards (Additional file 1: Figure S4). Biodistribution in target organs was followed over a period of 4 months following i.v. injection of 1.2 mg/kg SWCNT-ANXA5 in healthy mice (Fig. 4a, b). In contrast, the accumulation of SWCNT-ANXA5 in EMT6 tumor bearing mice following administration, as per the previously described injection protocol, was determined at 3 h following administration. (The time at which photothermal therapy was performed in the treatment study.) During the 4-month period preceding organ harvest, mice were monitored for physical side effects and abnormal behavior. No side effects were observed in any mice injected with SWCNT-ANXA5 during this time period. No histopathologic toxicity was observed during examination of hematoxylin and eosin stained FFPE sections from target organs at the conclusion of the study (Additional file 1: Figure S5).

## Discussion

The data presented here provide support for the effectiveness of combining targeted photothermal therapy and immune checkpoint inhibition for the treatment of metastatic breast cancer. This phenomenon is known as the “abscopal effect,” which describes the ability of localized radiation to initiate an antitumor response that suppresses tumor growth distant to the primary target. As early as the 1950s, researchers observed that localized tumor irradiation had a significant effect on distant tumors [22]. Studies have established that the systemic nature of the abscopal effect is due to the host’s immune response [23–25]. While γ-irradiation has been the prime focus of most abscopal research, a growing body of work demonstrates that photothermal therapy can induce an abscopal effect as well. Numerous studies have demonstrated that thermal ablation in combination with the checkpoint inhibitor anti-CTLA-4, produces a heightened immune response [16, 26–29]. We observe a similar abscopal response following targeted photothermal ablation and anti-CTLA-4 blockade in the EMT6 model of breast cancer.

The abscopal effect is illustrated by the data in Fig. 2a, b. Orthotopic EMT6 tumors grow rapidly and have metastasized by the time they are treated by photothermal therapy. This is the reason why mice with tumors treated only with photothermal therapy had only a slight increase in survival compared to the untreated mice control, even though the primary tumor had been completely ablated. The administration of anti-CTLA-4 alone delayed tumor growth compared to the control and increased survival time to 68 days but did not lead to cure. A similar result of delayed EMT6 tumor growth was observed by Jure-Kunkel et al. when anti-CTLA-4 was administered [30]. For the combination of photothermal therapy and anti-CTLA-4, 55% of the mice treated survived at 100 days after tumor inoculation and are likely cured.

Insight into the mechanism of antitumor immunity was evaluated by using flow cytometry to quantify the populations of immune effector cells in the spleen. Compared to either photothermal therapy or anti-CTLA-4 alone, the combination therapy resulted in sevenfold increase in helper CD4^+^ T cells and a threefold increase in cytolytic CD8^+^ T cells, both of these results being highly statistically significant (*p* < 0.005). These results provide further evidence of a potential abscopal response by the combination of phototherapy and T cell co-stimulation therapy with immune checkpoint inhibition.

Increases in CD4^+^ and CD8^+^ cell counts correlated with increases in spleen sizes observed during necropsy. Increased spleen size indicates a heightened immune response. The spleen is comprised of multiple cell types, the most common of which are of the CD4^+^, CD8^+^, and B-cell lineages [31, 32]. While not explored in this body of work, we would expect B-cell counts to increase along with CD4^+^ and CD8^+^ T cells counts [33]. Helper CD4^+^ T cells assist in humoral immunity by facilitating other immune cells by cytokine stimulation and direct cell–cell interactions. Cytolytic CD8^+^ T cells directly kill tumor cells. Increases in CD4^+^ and CD8^+^ cell counts are indicative of a systemic abscopal immune response.

The presence of a systemic abscopal immune response after the combination treatment is further supported by increases in mouse serum pro-inflammatory cytokine levels (Fig. 3). TNF-α activates tumor-associated macrophages to present antitumoral effects [34, 35]. IFN-γ plays an important role in tumor surveillance [36, 37]. IL-6 promotes the proliferation of macrophages and lymphocytes [38]. The significant increase in these effector molecules in mouse serum 7 days after PTT treatment combined with anti-CTLA-4 further supports the existence of an antitumoral immune response.

At the conclusion of irradiation of mice that received SWCNT, the average maximum tumor temperature was 54 °C; this temperature was sufficient to completely ablate the tumor (Fig. 1). This is within the 45–60 °C range in which enzyme inactivation and mitochondrial injury occurs [39]. The temperature of tumors in the saline control group remained below 40 °C, a temperature which provides minimal therapeutic benefit [39–41].

We observed the majority of SWCNT-ANXA5 accumulation based on concentration was primarily in the liver, heart, spleen, kidneys, lungs, and tumor (Fig. 4a). In mice with EMT6 tumors, we observed that the SWCNT-ANXA5 concentration was similar to that in the liver and kidneys (Fig. 4a, b). Trace amounts of SWCNT-ANXA5 were detected in the brain, large intestines, and small intestines. It is important to note that the biodistribution of SWCNT on the basis of % of injected dose (ID) does not correlate well with the absolute concentration of SWCNT within a target tissue. This is primarily due to the differences among organ weights. For instance, a given % ID within an organ will correspond to a higher concentration within smaller organs, and smaller concentrations within larger organs. Comparing samples on the basis of SWCNT concentration reveals that the highest concentration is within the kidney, followed closely by the liver and spleen (Fig. 4a).

There is some evidence of degradation of SWCNTs in the various organs over the period of the biodistribution study (Fig. 4a, b). Degradation of SWCNTs in organs is expected based on a previous finding that multi-walled carbon nanotubes are degraded in macrophages [42]. The SWCNTs used in our study have an average diameter of 0.8 nm and average length of 1500 µm. This size is not predicted to be cytotoxic based on a study by Zhu et al. [43], who classified the potential for lipid bilayer damage by carbon nanotubes toxicity based on length and diameter. According to this classification, the SWCNTs we used are in the “biologically soft” category, minimizing cytotoxicity, which is consistent with our observations in the studies in mice where we did not observe any side effects or histopathologic toxicity as a result of administering the SWCNT-ANXA5 conjugate.

## Conclusions

Here, we demonstrate a novel combinatorial treatment modality where we obtain a relatively high survival rate in mice with aggressive metastatic breast cancer using photothermal therapy with the SWCNT-ANXA5 bioconjugate combined with anti-CTLA-4-based checkpoint inhibition. The use of the tumor vascular targeting protein ANXA5 minimized the amount of systemically delivered SWCNT necessary to eradicate primary tumors in a low one-time dose. Interestingly, we noted an increase in the survival of mice with metastatic cancer treated with combinatorial therapy, even though only the primary tumor was irradiated. A mechanistic study quantifying the numbers of important splenic antitumor effector cells revealed that only the combination of both treatment modalities increased the number of CD4^+^ helper and CD8^+^ cytotoxic T cells. We hypothesize that this increase in T cells reflects an abscopal response, where antitumoral effector cells suppressed tumor metastasis. Although SWCNTs were still found to be present in organs 4 months after administration, no side effects or apparent tissue toxicity were observed during the course of experiments.

## Supplementary Information

**Additional file 1: Figure S1**. Characteristic emission of serial dilutions of (6,5) CoMoCAT SWCNTs in 1% SDS. **Figure S2** Raman spectra of SWCNT-ANXA5 (A) in phosphate buffered saline demonstrating characteristic G and D bands intrinsic to purified CNTs, (B) Bradford standard for determining the ANXA5 content of the ANXA5-SWCNT bioconjugate, and (C) UV-VIS standard at 808 nm for quantification of SWCNT content in the bioconjugate. **Figure S3** Relative percentages and counts for splenic autitumor immune effector cells by flow cytometry analysis. **Figure S4** Relative fluorescence units (RFU) as a function of SWCNT concentration in ex situ tissue lyase at four excitation wavelengths. **Figure S5** Histopathology of target organs in 3 mice 4-months following i.v. injection of 1.2 mg/kg SWCNT-ANXA5 in healthy BALB/cj mice.

## Data Availability

All data are fully available without restriction.

## Abbreviations

SWCNT: Single-walled carbon nanotube
ANXA5: Annexin A5
anti-CTLA-4: Anti-cytotoxic T-lymphocyte-associated protein 4
EDTA: Ethylenediamine tetraacetic acid
SDS-PAGE: Sodium dodecyl sulfate–polyacrylamide gel electrophoresis
DSPE: 1,2-Distearoyl-sn-glycero-3-phosphoethanolamine
PTT: Photothermal therapy
