# Supplementary material for: Targeted Single-Walled Carbon Nanotubes for Photothermal Therapy Combined with Immune Checkpoint Inhibition for the Treatment of Metastatic Breast Cancer
Source: Nanoscale Res Lett. 2021 Jan 7;16:9. doi: 10.1186/s11671-020-03459-x (PMC7790975; doi:10.1186/s11671-020-03459-x)
Supplement: Supplementary file 1 — Additional file 1: Figure S1. Characteristic emission of serial dilutions of (6,5) CoMoCAT SWCNTs in 1% SDS. Figure S2 Raman spectra of SWCNT-ANXA5 (A) in phosphate buffered saline demonstrating characteristic G and D bands intrinsic to purified CNTs, (B) Bradford standard for determining the ANXA5 content of the ANXA5-SWCNT bioconjugate, and (C) UV-VIS standard at 808 nm for quantification of SWCNT content in the bioconjugate. Figure S3 Relative percentages and counts for splenic autitumor immune effector cells by flow cytometry analysis. Figure S4 Relative fluorescence units (RFU) as a function of SWCNT concentration in ex situ tissue lyase at four excitation wavelengths. Figure S5 Histopathology of target organs in 3 mice 4-months following i.v. injection of 1.2 mg/kg SWCNT-ANXA5 in healthy BALB/cj mice. [file 11671_2020_3459_MOESM1_ESM.docx]

**Supplementary Information**


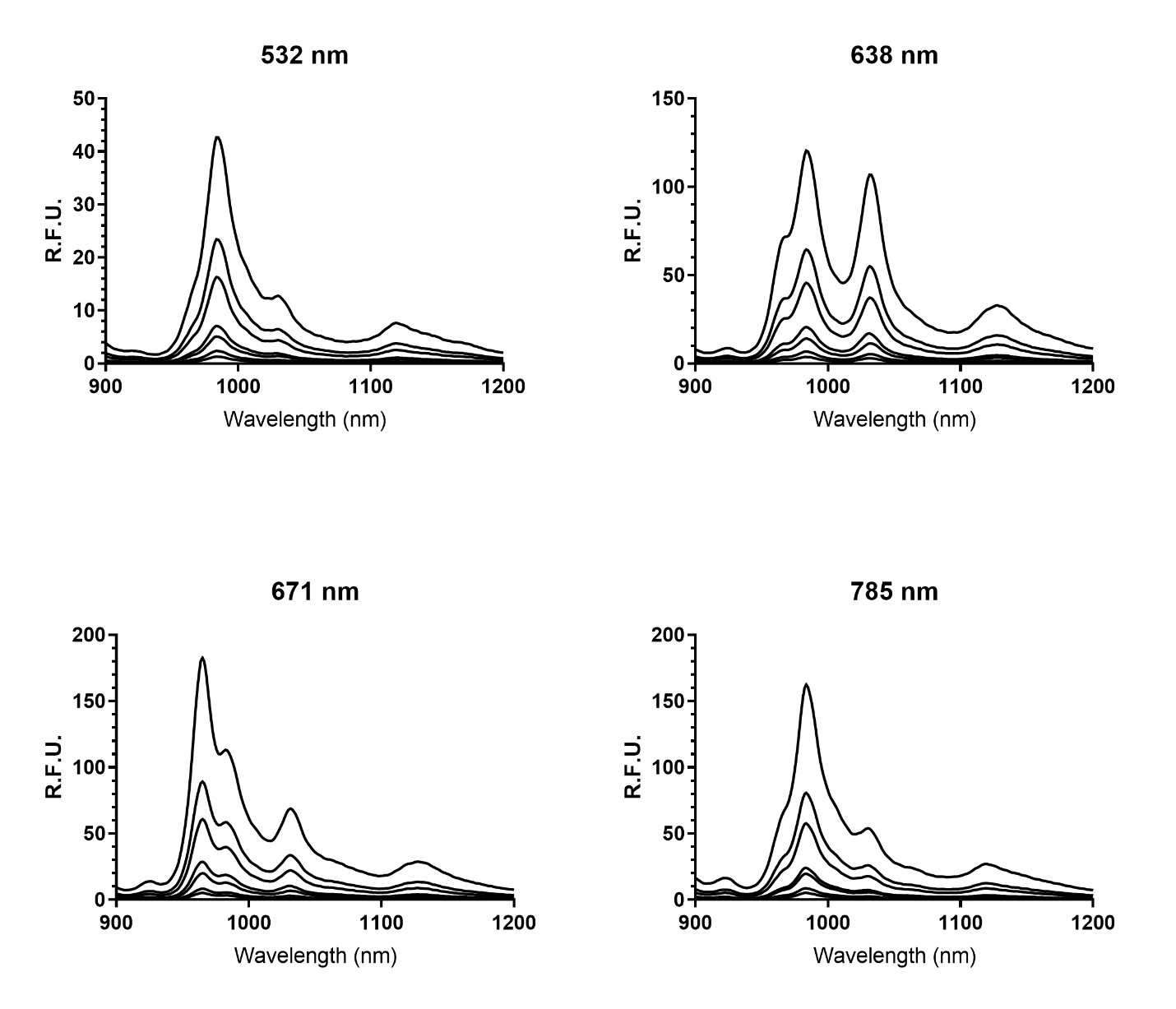


**Figure 1.** Characteristic emission of serial dilutions of (6,5) CoMoCAT SWCNTs in 1% SDS after stimulation with 532, 638, 671 and 785 nm visible light - near infrared spectroscopy. The initial concentration was 42 mg/L, and each dilution was 1:2.


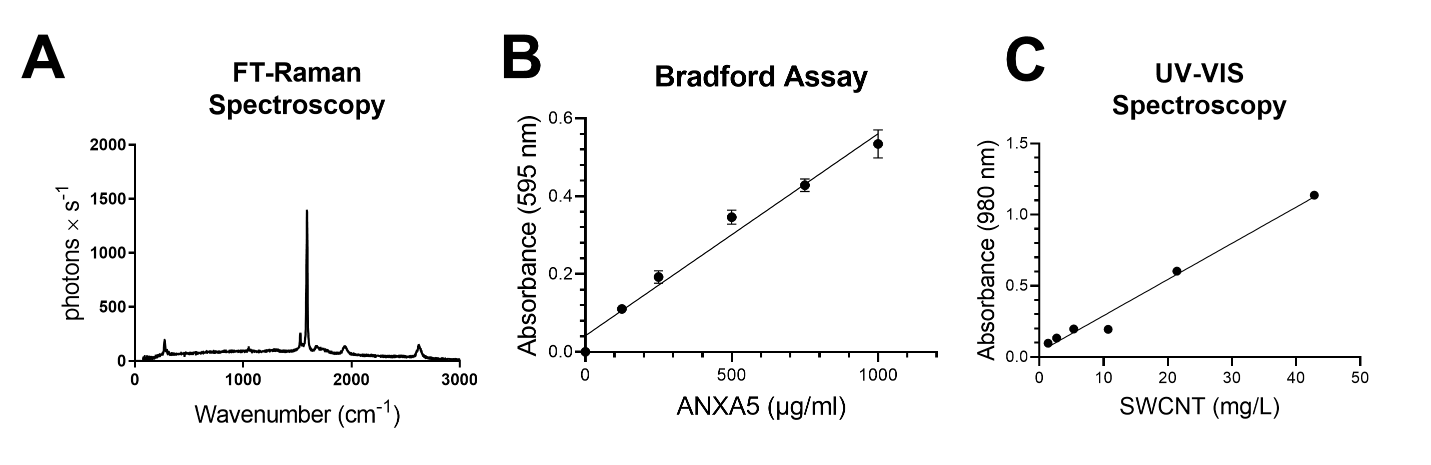


**Figure 2.** Raman spectra of SWCNT-ANXA5 (A) in phosphate buffered saline demonstrating characteristic G and D bands intrinsic to purified CNTs, (B) Bradford standard for determining the ANXA5 content of the ANXA5-SWCNT bioconjugate, and (C) UV-VIS standard at 808 nm for quantification of SWCNT content in the bioconjugate.

**Figure 3.** Relative percentages (A) and counts (B) for splenic autitumor immune effector cells by flow cytometry analysis.

**Figure 4.** The detection of SWCNTs in *ex situ* tissue lyase can be accomplished by fluorescent spectroscopy at several excitation spectra. Using this fluorescence, biodistribution of SWCNT-ANXA5 measured as % of injected dose and tissue concentration in g/L was determined. R.F.U.: relative fluorescence units


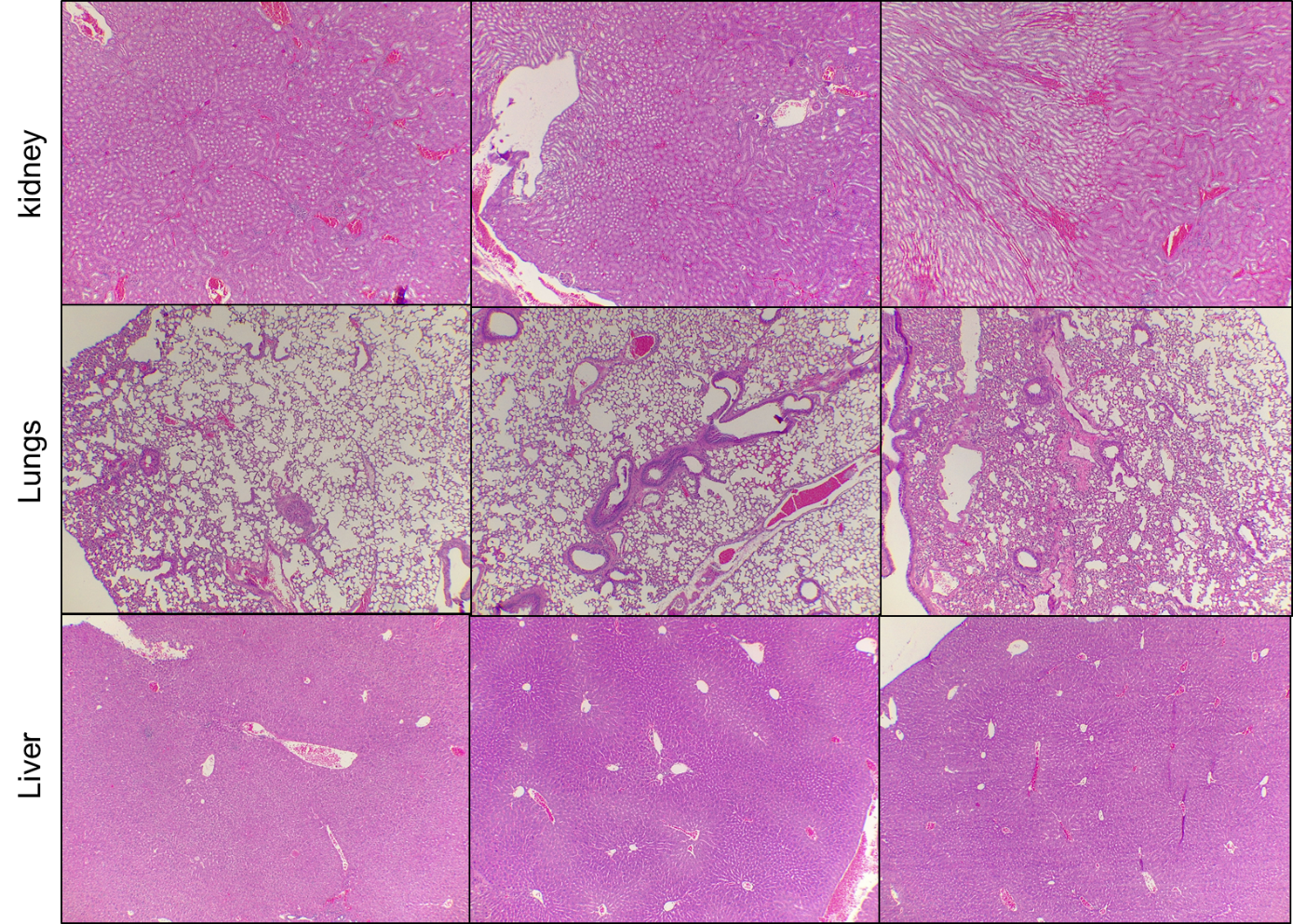


**Figure 5.**  Histopathology of target organs in 3 mice 4-months following a i.v. injection of 1.2 mg/kg SWCNT-ANXA5 in healthy BALB/cj mice. No organ specific cytotoxicity was observed.
